# The BTB/POZ zinc finger protein Broad-Z3 promotes dendritic outgrowth during metamorphic remodeling of the peripheral stretch receptor dbd

**DOI:** 10.1186/1749-8104-6-39

**Published:** 2011-12-12

**Authors:** Janet A Scott, Darren W Williams, James W Truman

**Affiliations:** 1 Department of Biology, Box 351800, University of Washington, Seattle, WA 98195, USA; 2 Janelia Farm Research Campus, Howard Hughes Medical Institute, 19700 Helix Drive, Ashburn, VA 20147, USA; 3 MRC Centre for Developmental Neurobiology, King's College London, Guy's Hospital Campus, London SE1 1UL, UK

## Abstract

**Background:**

Various members of the family of BTB/POZ zinc-finger transcription factors influence patterns of dendritic branching. One such member, *Broad*, is notable because its BrZ3 isoform is widely expressed in *Drosophila *in immature neurons around the time of arbor outgrowth. We used the metamorphic remodeling of an identified sensory neuron, the dorsal bipolar dendrite sensory neuron (dbd), to examine the effects of BrZ3 expression on the extent and pattern of dendrite growth during metamorphosis.

**Results:**

Using live imaging of dbd in *Drosophila *pupae, we followed its normal development during metamorphosis and the effect of ectopic expression of BrZ3 on this development. After migration of its cell body, dbd extends a growth-cone that grows between two muscle bands followed by branching and turning back on itself to form a compact dendritic bundle. The ectopic expression of the BrZ3 isoform, using the GAL4/UAS system, caused dbd's dendritic tree to transform from its normal, compact, fasciculated form into a comb-like arbor that spread over on the body wall. Time-lapse analysis revealed that the expression of BrZ3 caused the premature extension of the primary dendrite onto immature myoblasts, ectopic growth past the muscle target region, and subsequent elaboration onto the epidermis. To control the timing of expression of BrZ3, we used a temperature-sensitive GAL80 mutant. When BrZ3 expression was delayed until after the extension of the primary dendrite, then a normal arbor was formed. By contrast, when BrZ3 expression was confined to only the early outgrowth phase, then ectopic arbors were subsequently formed and maintained on the epidermis despite the subsequent absence of BrZ3.

**Conclusions:**

The adult arbor of dbd is a highly branched arbor whose branches self-fasciculate to form a compact dendritic bundle. The ectopic expression of BrZ3 in this cell causes a premature extension of its growth-cone, resulting in dendrites that extend beyond their normal muscle substrate and onto the epidermis, where they form a comb-shaped, ectopic arbor. Our quantitative data suggest that new ectopic arbor represents an 'unpacking' of the normally fasciculated arbor onto the epidermis. These data suggest that the nature of their local environment can change dendrite behavior from self-adhesion to self-avoidance.

## Background

Neuronal development requires the coordination of fate specification, cell migration, neurite pathfinding, arbor elaboration, terminal field refinement and synaptogenesis. Understanding how these dynamic developmental processes give rise to the diverse cellular forms and specific patterns of connectivity of the nervous system is a primary goal of developmental neuroscience. In comparison to the attention given to axon pathfinding and synaptogenesis, relatively little is known about the cellular dynamics of dendritic arborization, with few examples of *in vivo *studies in intact animals [1-4].

The multidendritic (md) sensory neurons of *Drosophila melanogaster *are a well-established model system for examining the development and maintenance of dendritic shape [5], and are accessible for time-lapse imaging in intact animals [6-9]. The dorsal cluster of md neurons includes the dorsal bipolar dendrite (dbd) neuron, a conserved stretch receptor that is found in insects from silverfish to moths [10]. While the embryonic development of dbd has been described in detail [10-13], little is known about the development of its adult dendritic arborization. In this study we describe the first *in vivo *observations of the development of a fasciculated dendritic arbor such as is found in dbd.

Our previous work has revealed how developmental hormones are important for orchestrating the metamorphic development of the sensory system in *Drosophila *[3,14]. Here we used dbd to investigate the effects of the Z3 isoform of the BTB/POZ zinc-finger transcription factor Broad (BrZ3) on dendritic development in intact animals. Broad is best known for transducing 20-hydroxyecdysone signals into transcriptional cascades at metamorphosis [15-17]. In *D. melanogaster*, the four Broad splice variants have unique carboxy-terminal zinc-finger pairs (Z1 to Z4), which serve tissue-specific independent, partially redundant, and combinatorial functions [16]. The rbp, br, and 2Bc complementation groups, attributed to BrZ1, Z2, and Z3, respectively, are all pupal lethal, and npr flies with mutations in the Broad core domain die before pupariation [18]. The rbp, br, and 2Bc mutants all fail to complete metamorphosis of the central nervous system (CNS), with additional defects in optic lobe neuropil morphogenesis seen with br (BrZ2) and 2Bc (BrZ3) alleles [19]. BrZ3 is the predominant Broad isoform in CNS neurons [20-22]. In wandering larvae, BrZ3 is highly expressed in photoreceptors and the immature adult-specific interneurons throughout the CNS [22,23]. However, unlike Broad isoform expression in other tissues, which has a 'pupal specification' function [24], the expression of BrZ3 in central neurons is uncoupled from metamorphosis and marks a distinctive, early phase in the developmental maturation of central interneurons, regardless of the life-stage when they are born [22]. BrZ3 expression occurs after the completion of pathfinding as young neurons are initiating arbor outgrowth, but it then disappears when arbor elaboration is well underway [22].

The roles of the temporal and spatial patterns of BrZ3 expression in developing neurons are poorly understood. Widespread misexpression of BrZ3 in neurons using the *elav-GAL4 *driver or in glia using *repo-GAL4 *was embryonic lethal (data not shown). However, BrZ3 function has rarely been examined in identified neurons. Here we used time-lapse imaging of dendrites of dbd to investigate how BrZ3 affects neuronal development in real time. We found that with BrZ3 misexpression, dbd extended beyond its appropriate muscle substrate onto the epidermis, and in this new environment, its dendrites changed from having a robust fasciculated architecture to a tree-like, self-avoidance pattern of arborization. This phenotype is correlated with a premature extension of dendrite growth cones, suggesting that Broad expression can modulate the timing of dendritogenesis.

## Results

### The remodeling of the dendritic arbor of dbd during metamorphosis

**Figure 1:**
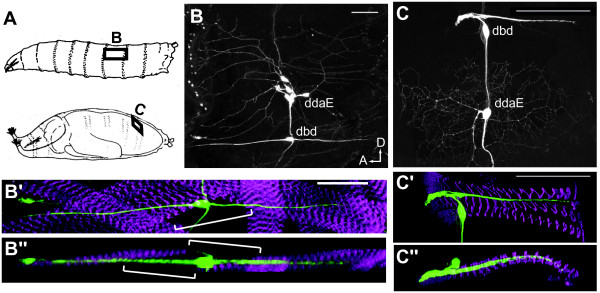
**The dendritic arborizations of the dorsal bipolar dendrite neuron (dbd) in larval and pharate adult *Drosophila***. **(A) **Cartoon of the segmental position of dbd before and after metamorphosis. **(B, C) **Dorsal views of a dorsal abdominal segment from a larva and a pharate adult, respectively, showing the position of dbd relative to the md sensory neuron, ddaE. (B'-C") Confocal stacks from a larva and a pharate adult projected along the Z- axis (B', C') and the Y-axis (B", C") showing the relationship of the stretch receptor to the surrounding muscles; green, anti-CD8; magenta, phalloidin F-actin. (B', B'') Larval version of dbd; brackets show clearest region of larval dbd arbor unassociated with muscles. (C', C'') In the adult version of dbd, the dendrites remained within the dorsal abdominal muscle layer. Scale bar = 50 μm. In all figures, dorsal is up and anterior is left. Genotype is *C161-GAL4, UAS mCD8::GFP*.

Like other classes of md neurons, the dendritic arbor of dbd is completely remodeled during metamorphosis. In its larval form, dbd has a single, bipolar dendrite that spans an abdominal segment within the second superficial muscle layer beneath the dorsal epidermis (Figure 1A-B''; see also [10]). Unlike the simple bipolar dendrite in the larva, the adult form of the cell has a densely fasciculated dendritic bundle that fills the space between two of the dorsal longitudinal muscle fibers that extend from midsegment to the posterior segment border (Figure 1C-C''). These dorsal abdominal muscles are retractors of the abdominal tergites [25], and a single dbd serves as an internal stretch receptor in the set of 15 to 25 muscles per hemi-segment. The larval muscles, with which dbd makes contact, degenerate early in metamorphosis and are replaced by adult-specific muscles. Persistent twist-expressing myoblasts migrate to the dorsal abdomen and fuse, generating a new substrate for dbd to associate with [26]. Unlike the larval dbd, which only indirectly contacts muscles at the distal tips of dendrites [10], the adult-specific dendrites of dbd appear to make direct contact with their muscle substrate at multiple points along its length (data not shown). We followed the fate of dbd by using the *C161-GAL4 *driver that expresses in a subset of the multidendritic sensory neurons, including dbd [27]. Using the appropriate upstream activation sequence (UAS) constructs, we could then selectively express mCD8::GFP and/or broad isoforms in these cells.

### Initial outgrowth of the adult dbd dendrites

**Figure 2:**
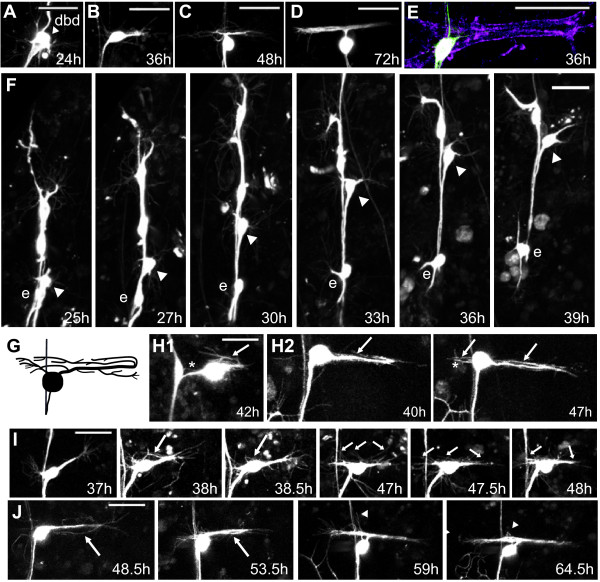
**Development of the fasciculated dbd arbor**. **(A-D) **Representative *in vivo *confocal z-projections of dbd migration and arbor formation from 24 h to 72 h APF. **(E) **Initial outgrowth onto immature myoblasts: green, anti-CD8; magenta, phalloidin F-actin. **(F) **Z-projections from two-photon time-lapse videos showing dbd (arrowhead) and neuron ddaE (e) between 25 h and 39 h APF. **(G) **Cartoon of the branches within the dbd arbor. **(H-J) **Time-lapse z-stacks showing four features of dbd branching and elaboration. (H1) and (H2) show posterior growth cone reversal (arrows), and new branches from the soma (asterisks). (I, J) Arbor thickened through adhesion of growth cone filopodia (large arrows), and interstitial filopodia (small arrows), with occasional branch pruning (arrowheads). Scale bar = 30 μm.

Two-photon image stacks were taken through the abdominal body wall of animals at various times during the approximately 100 h from puparium formation until adult emergence. The larval dendrites of dbd were pruned back by 24 h after puparium formation (APF), the cell body migrated dorsally by 36 h APF, and generated its adult-specific dendritic tree between 36 and 72 h APF (Figure 2A-D; n ≥ 4 animals per time point).

**Figure 3:**
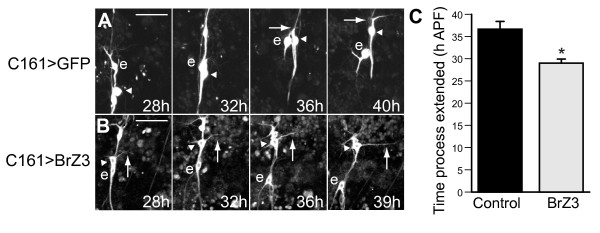
**Outgrowth of dbd dendrites**. Z-projections from two-photon time-lapse videos showing dbd (arrowhead) and neuron ddaE (e) between 28 h and 40 h APF. **(A) **Control migrating dbd (arrowheads) extended a large growth cone (arrow) along the nerve, passed ddaE (e), ceased migration, then began posterior outgrowth by 40 h APF. **(B) **In neurons expressing BrZ3 (dbd^[+BrZ3]^), outgrowth began before migration was complete (arrows). **(C) **Time of initiation of dendrite outgrowth, using the metric of reaching twice the length of the growth cone. Control, 36.6 ± 1.8 h APF (mean ± standard error of the mean), n = 4; dbd^[+BrZ3]^, 29.4 ± 0.9 h APF, n = 9; *P = 0.0017. Scale bar = 30 μm.

To gain insight into the cellular dynamics of dendritogenesis, we performed time-lapse imaging at 30-minute intervals from 24 h to 45 h APF and observed the sequential steps of pruning, soma migration, and directed outgrowth (Figures 2F and 3A; Additional file 1). After pruning, the dbd cell body began to migrate dorsally along the peripheral nerve (Figures 2F and 3A; see also [3]). During migration, dbd extended one or, occasionally, two large growth cones that appeared to pull the cell body dorsally along the nerve, passing the dendritic arborizing neuron ddaE migrating in the opposite direction. Growth cones were oriented dorsally, anteriorly, and/or posteriorly during migration. Migration ended between 32 h and 38 h APF (mean = 36 h APF, n = 6). Within one hour after completing migration, a growth cone began extending posteriorly along the developing muscle to establish the primary dendrite. An occasional second anterior-directed growth cone also began extension around this time, but this branch was not usually maintained to the adult. The mean time when primary dendrite extended to twice the length of the exploratory growth cone was 37 h APF (Figure 3C; n = 4). This dendrite extended onto immature, spreading myoblasts, while myopodia were still visible at the ends of muscles, and prior to Z-band formation (Figure 2E).

### Adult arbor elaboration in dbd

Time-lapse imaging revealed four modes of dendritic growth that contributed to the formation of the distinctive dendritic arbor of dbd: (1) extension of the primary dendrite from the cell body (Figure 2H1, H2, J), (2) branch foldback from the posterior boundary toward the anterior (Figure 2H1, H2, J, arrows), (3) filopodial adhesion onto the primary neurite from growth cones and bundle termini (Figure 2I, large arrows), and (4) incorporation of interstitial filopodia (Figure 2I, small arrows) (Additional file 2).

During primary dendrite extension, we observed a posterior 'pull' on the dbd cell body most often between 40 and 45 h APF (Figure 2I, 37 to 38.5 h). This pull, visible as a sudden jerk of the cell body for one to three frames in the time-lapse series, often separated the cell body from its close association with the peripheral nerve and dislodged whatever filopodia or branchlets that had been anterior to the cell body (n = 6). Following this pull, dendrite outgrowth was observed from the posterior boundary back toward the anterior, closely apposed with the initial dendrite in the muscle groove (n = 7). We could not always resolve if the anteriorly advancing dendrite arose from growth cone turning or from a new branch from the growth cone (for example, Figure 2I). However, in three movies we directly observed that the posterior growth cone turned 180° and grew back anteriorly. In a few cases it was clear that this reflected growth cone eventually extended anterior to the cell body (Figure 2H2, arrows). This folding back of the dendrite at the posterior boundary was also revealed by staining for the MAP1B-like protein Futsch [28]. Within some dbd arbors, this microtubule marker labeled dendrites that reached from the soma to the posterior boundary, then bent and returned to the anterior (data not shown). In mature arbors, this Futsch staining showed that these microtubule-containing branches form the core of the dendrite bundle (data not shown).

The dendritic arborization of dbd thickened through the addition of filopodia from terminal and interstitial branch sites. During initial outgrowth, some of the exploratory filopodia from growth cones adhered back onto the primary neurite (Figure 2I, J, large arrows). The posterior arbor boundary remained a dense zone for filopodial activity throughout elaboration. Interstitial filopodial activity intensified starting between 40 and 45 h APF and ending between 56 and 60 h APF (Figure 2I, 47 to 48 h, small arrows; n = 7). Interstitial filopodia first extended perpendicular to the bundle, then tilted toward the anterior or the posterior and adhered, visibly increasing the caliber of the dendrite bundle (Figure 2I, J). Both interstitial filopodia and new primary branches from near the cell body contributed to the anterior branch. In control dbd neurons, higher-order branches within the dendrite bundle were too densely packed to be observed in detail. However, the transition from longer to shorter and sparser filopodia at approximately 60 h APF (n = 3) suggested that dbd branches may have had some tertiary branching. Refinement of the posterior arbor after 60 h APF was subtle, but filopodial activity anterior to the cell body was still evident up to 65 to 70 h APF (Figure 2J, 64.5 h).

### BrZ3 expression transforms dendritic arbor shape

**Figure 4:**
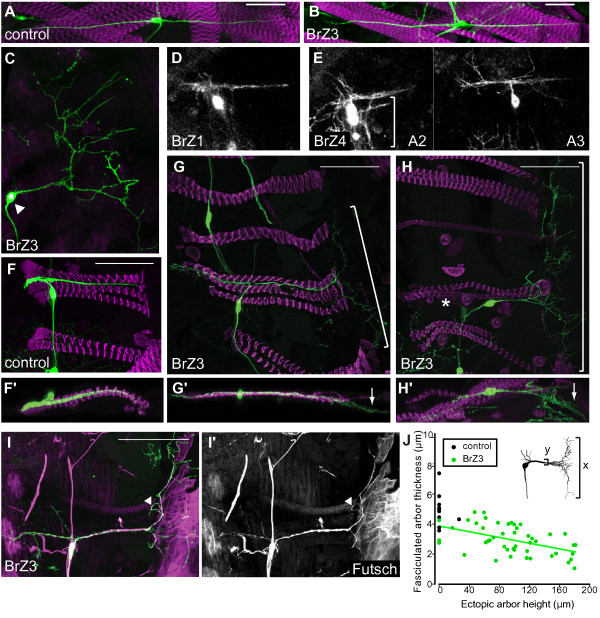
**Ectopic BrZ3 expression generated a stereotypic ectopic arbor in the adult dbd neuron during metamorphosis**. **(A, B) **Morphology of the dendritic arbor of the dbd neuron in the last larval stage of controls (A: C161-GAL4 > UAS mCD8::GFP) and individuals expressing BrZ3 (B: C161-GAL4 > UAS mCD8:GFP, UAS-BrZ3 dbd [dbd^[+BrZ3]^]). **(C-E) **The effects of expressing different Broad isoforms in dbd on its dendritic morphology at the end of metamorphosis. (C) Pharate adult morphology of dbd^[+BrZ3]^; arrowhead, anti-Broad-Core immunostaining. (D) Morphology of dbd^[+BrZ1] ^is similar to control. (E) Morphology of dbd^[+BrZ4] ^was variable but typically maintained contact with muscles. Bracket: dbd arbor in adjacent muscle grooves. **(F) **Control pharate adult dbd. **(F') **Y-projection. **(G, H) **Examples of dbd^[+BrZ3] ^ectopic combs (brackets) posterior to dorsal abdominal muscles. Loss of anterior dendrites (asterisk) occurred in 25% of dbd^[+BrZ3] ^neurons. **(G', H') **Y-projections showing the ectopic comb (arrows) was superficial to the muscle layers, veering toward the cuticle. **(I, I') **MAP1B-like antibody labeled microtubules in the ectopic arbor 'backbone' and some branches (arrowhead): green, anti-CD8; magenta, Futsch; 50 h APF. **(J) **Control neurons (black) had thicker dendritic bundles than dbd^[+BrZ3] ^neurons (green). The trend line shows a significant negative correlation between dorsal-ventral ectopic arbor height and main bundle thickness among dbd^[+BrZ3] ^(r = -0.49, *P *< 0.01). Scale bar = 50 μm. Immunostaining except for (I): green, anti-CD8; magenta, phalloidin.

We examined the effects of expressing Broad isoforms on the growth of dbd, and found the Z3 isoform of Broad (BrZ3) transformed the shape of the adult dbd dendrites from its normal bipolar form into a tree-like arborization. In the larva, BrZ3 misexpression did not interfere with the dendritic morphology of dbd or its association with target muscles (Figure 4A, B; n = 15 neurons in 4 animals). However, during metamorphosis, GAL4^C161^/UAS-induced expression of BrZ3 resulted in a stereotyped, comb-like arborization in an ectopic position (Figure 4C; n = 45 of 48 neurons). The ectopic arborization was unique to dbd neurons expressing the BrZ3 isoform (dbd^[+BrZ3]^). The dbd^[+BrZ1] ^neurons were normal (Figure 4D; n = 18 neurons in 7 animals). The dbd^[+BrZ4] ^neurons were highly variable from segment to segment (Figure 4E; n = 31 neurons in 9 animals) and showed multiple short branches that extended from the muscle groove, but they did not consistently produce a stereotyped ectopic arbor. Staining for muscles showed that control arbors did not leave the muscle (Figure 4F), but the dbd^[+BrZ3] ^ectopic arbor extended beyond the posterior ends of the muscles and onto the epidermis (Figure 4G-H'; n = 39 of 42 neurons). Immunostaining against the microtubule-associated protein Futsch showed that that the major axis of the dbd^[+BrZ3] ^ectopic arbor, the comb 'backbone', and some higher-order branches were stabilized with microtubules by 50 h APF (Figure 4I, arrowhead; n = 6). While the general shape and orientation of this comb-like arbor were consistent, its footprint ranged in dorsal-ventral axis (height) from 18 to 181 μm (mean = 97 μm, standard deviation = 49), and in the anteroposterior axis (length) from 1 to 90 μm (mean = 42 μm, standard deviation = 19). Several measures suggest that dbd^[+BrZ3] ^dendrites spread onto the epidermis at the expense of the fasciculated main arbor. Overall, the main dendritic bundle of dbd^[+BrZ3] ^was thinner than in control cells (Figure 4J; *P *= 0.0002, *t*-test assuming unequal variances). Within the range of dbd^[+BrZ3] ^morphologies, larger ectopic arbor size was correlated with decreased thickness of the fasciculated arbor (Figure 4J; r = -0.49, *P *< 0.01). Also, failure of the normal arborization to reach the anterior margin of the muscle was correlated with a larger ectopic arbor size (Figure 4H, asterisk; r = 0.29, *P *= 0.023). Taken together, these measurements suggest that the dendrites of dbd^[+BrZ3] ^are primarily misplaced rather than overgrowing.

### BrZ3 expression results in premature dendrite outgrowth

**Figure 5:**
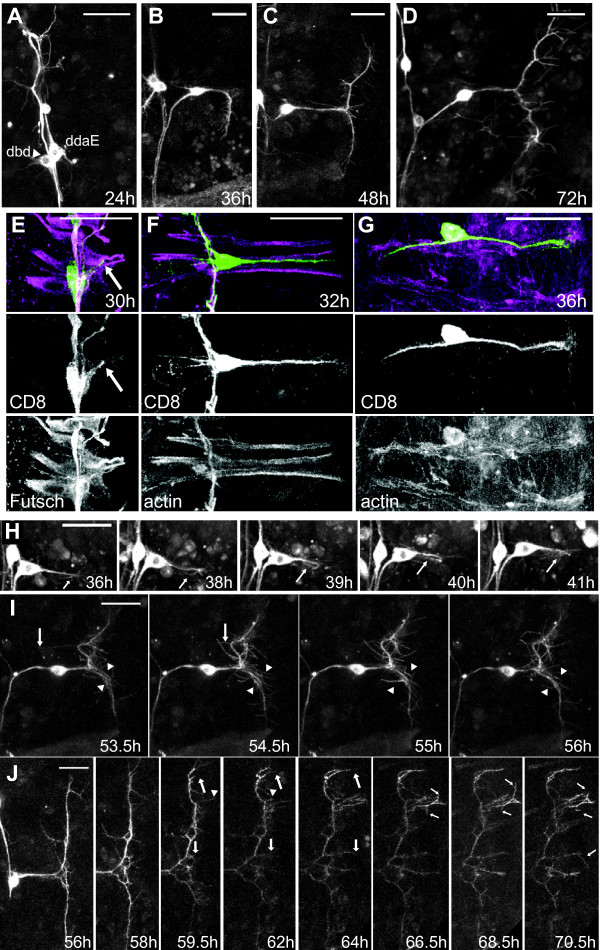
**Elaboration of the BrZ3 ectopic arbor**. **(A-D) **Representative *in vivo *confocal z-projections of dbd^[+BrZ3] ^at various times during metamorphosis. **(E-G) **Confocal z-projections of dbd^[+BrZ3] ^at 30, 32, and 36 h APF showing the relationship of the extending dendrite to the underlying myoblasts. Green, anti-CD8; magenta, anti-Futsch (E) or phalloidin F-actin (F, G). The muscle groove in (G) was stretched apart in preparation. Dbd growth cones (arrows). **(H-J) **Videos of branching in dbd^[+BrZ3]^. (H) Fasciculation through growth cone reversal (arrows). (I) The rare ectopic branches that attempted to fasciculate were removed (arrowheads), as were branches growing into the next muscle groove (small arrows). (J) Rapid extension of secondary branches (large arrows) followed by limited stabilization of tertiary filopodia (small arrows). Significant branches can be retracted (small arrowheads). All times are hours APF. Scale bar = 30 μm.

To determine the developmental origins of this ectopic arbor, we examined the time-course of dbd^[+BrZ3] ^development *in situ *throughout pupal development and compared this to growth of control neurons. The pruning of larval dendrites occurred normally (Figures 3 and 5A; n = 15 neurons in 4 animals), but dbd^[+BrZ3] ^consistently exhibited early dendritic outgrowth. The earliest time at which dbd^[+BrZ3] ^initiated growth cone extension was 24 h APF, and by 30 h APF all dbd^[+BrZ3] ^were showing outgrowth with an apparently normal trajectory (n = 15 neurons in 4 animals, data not shown). By 36 h APF, dbd^[+BrZ3] ^had significantly longer projections (mean = 41 μm, standard error of the mean = 3 μm, n = 19 neurons in 7 animals) than did control dbd (mean = 20 μm, standard error of the mean = 2 μm, n = 23 neurons in 6 animals; Student's *t*-test *P *< 0.00001). Immunostaining for muscles showed that this premature outgrowth occurred along muscles that had not yet grown to their full length (Figure 5E, F).

We examined the effect of BrZ3 expression on the timing of outgrowth in more detail using time-lapse imaging (Figure 3; Additional files 3, 4, and 5). During migration, both dbd and dbd^[+BrZ3] ^extended a short growth cone process (< 14 μm), usually to the posterior. As dbd^[+BrZ3] ^neared its final location, the process shifted dorsoposteriorly and began rapid extension before soma migration was complete. This 'cutting the corner' of dbd's usual path led to early dendrite extension that, in most cases, became stabilized. Extension of a stable posterior primary dendrite to twice the length of the exploratory growth cone occurred in dbd^[+BrZ3] ^between 26 h and 33 h APF (Figure 3C; mean = 29 h APF), significantly earlier than controls (mean = 37 h APF; *P *= 0.0017, Student's *t*-test). In contrast, dbd^[+BrZ3] ^soma migration ended only slightly earlier (mean = 33 h APF) than controls (mean = 36 h APF; *P *= 0.017, Student's *t*-test). Therefore, premature dendrite extension in dbd^[+BrZ3] ^occurred while the soma was still migrating dorsally along the intersegmental nerve (Figure 3B, arrowheads; n = 13).

### Elaboration of the ectopic arbor caused by BrZ3 expression

In intact animals imaged at single time points, ectopic arbor was first seen at 36 h APF, as a 90 degree turn forming a 'T' perpendicular to the main arbor (Figure 5B; n = 7 of 19 neurons). Staining for muscles showed that this ectopic branching arose at the posterior boundary of the myoblast substrate (Figure 5G). By 48 h APF, all dbd^[+BrZ3] ^had formed this 'T' bend (Figure 5C; n = 15 of 18 neurons in 8 animals), which was never seen in age-matched controls (Figure 2C; n = 9 neurons in 5 animals). In 80% of these dbd^[+BrZ3] ^the 'T' had clearly extended onto the epidermis. Second-order branches extending posteriorly from the 'T' were first seen at 48 h APF, and were more extensive at 65 h and 72 h APF. By 72 h APF, 88% of the dbd^[+BrZ3] ^had an ectopic arbor and 25% lacked an anterior arbor (Figure 5D; n = 34 neurons).

In comparison to the densely packed dendritic arbor of the control dbd, the transitions between primary dendrite extension, and secondary and tertiary branching phases of growth were easier to see in dbd^[+BrZ3] ^time-lapse videos (Figure 5; Additional file 5). The backbone of the ectopic arbor, the 'T' branch perpendicular to the main arbor, usually formed between 30 and 40 h APF (n = 5 of 7) but could begin growth as late as 45 h APF (n = 2). The posterior 'pull' of the dbd soma was coincident with or just prior to initiation of the 'T' branch (n = 7). This 'T' shape usually formed in two steps. First, the primary growth cone failed to fold back at the end of the muscle, and instead turned 90° and grew dorsally or ventrally, leaving a dense filopodial zone at the turn point. A second perpendicular branch arose from this filopodial zone, completing the 'T' backbone. Small additions and retractions set the final 'T' size by 46 to 60 h APF. Only one time-lapse of a control dbd showed a 90° bend of the primary dendrite, but this short bend was removed within one hour.

Time-lapse sequences showed no obvious differences between control dbd and dbd^[+BrZ3] ^in the timing of the elaboration phase. Secondary branching consisted of filopodial activity along the length of the primary dendrite stabilizing into branches, including forming the 'teeth' of the comb along the 'T' branch (Figure 5I, J). Secondary branches in the ectopic arbor formed between 40 and 65 h APF (Figure 5J, large arrows) and the few higher-order branches formed between 55 and 70 h APF (Figure 5J, small arrows).

The features of fasciculation characteristic of control dbd neurons were also observed in the time-lapse videos of dbd^[+BrZ3]^, but only in segments of dendrites along the muscle and not in dendrites that were on the epidermis. Often the dendritic zone at the apex of the 'T' branch generated both foldback branches into the muscle groove and extensions onto the epidermis during the arbor elaboration phase (n = 9). These responses of different branches of the same cell showed that fasciculation was substrate-specific. In the muscle groove, dbd^[+BrZ3] ^dendrites fasciculated back onto the primary dendrite through growth cone turning (Figure 5H; n = 6) and filopodial adhesion (data not shown; n = 5). However, in some cells, fasciculation was reduced or absent because when the dbd^[+BrZ3] ^soma was pulled far to the posterior, few to no filopodia were found along the shortened main arbor trunk (Figure 5I). On the epidermis, dbd^[+BrZ3] ^secondary branches attempting to fasciculate onto the 'T' backbone were rarely observed and were removed within two hours (Figure 5I, arrowheads).

While filopodia were dynamic for hours before forming stable secondary branches (Figure 5J, arrowheads), we rarely observed pruning that reduced the ectopic arbor backbone. In one time-lapse sequence, the highly branched ventral side of the 'T' backbone was removed through a combination of pruning and fasciculation with the main arbor in the muscle groove (54 to 56 h APF, data not shown). In another video we observed complete removal of dbd^[+BrZ3] ^epidermal ectopic arbor; a small, late-forming ectopic 'T' backbone was rapidly trimmed back by 30 μm, condensing its branches into a small area between 47 and 50 h APF (Additional file 3). Therefore, dbd could occasionally recover from the errors generated by ectopic BrZ3 expression.

### Phenocritical period for ectopic arbor

**Figure 6:**
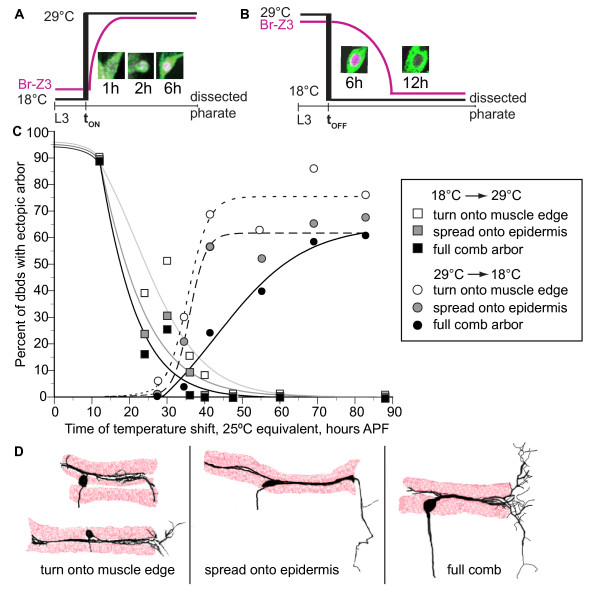
**Phenocritical window of BrZ3 action**. **(A) **Temperature step-up (t_ON_) from 18°C to 29°C removed Gal80^ts ^suppression to allow C161-GAL4 > UAS-BrZ3 expression within 2 to 3 h (2.3 to 3.5 h^25°C^). **(B) **Temperature step-down (t_OFF_) from 29°C to 18°C activated Gal80^ts^, removing BrZ3 expression by 12 h (approximately 6 h^25°C^). **(C) **Percent of pharate adult, dbd neurons with ectopic arbor after t_ON _(squares) and t_OFF _(circles) steps, converted to equivalent hours APF at 25°C. Cumulative percentages included three levels of ectopic arbor severity: comb arbor only (black), any microtubule-containing arbor on the epidermis (gray), and any turn along the posterior muscle edge (white). Best fit variable slope sigmoidal curves. t_ON_: 12 h, n = 10; 24 h, n = 92; 30 h, n = 39; 36 h, n = 116; 40 h, n = 73; 48 h, n = 62; 60 h, n = 26; 88 h, n = 60. t_OFF_: 29 h, n = 16; 34.5 h, n = 76; 41.4 h, n = 74; 55 h, n = 65; 69 h, n = 29; 83 h, n = 59. **(D) **Cartoons of the three grades of arbor phenotypes drawn from representative confocal images. The mildest phenotype category included dbds with dendritic arbor bending out of the muscle groove, and extending short, fine branches near the muscle. The intermediate category included longer branches that spread further onto the epidermis and were stabilized with microtubules. Full arbors had microtubule-containing large arbors on the epidermis with secondary branching.

To determine what temporal window of BrZ3 action generated the dbd ectopic arbor, we systematically delayed the onset of BrZ3 expression with a temperature-sensitive allele of the GAL4 suppressor GAL80 (GAL80^ts^), and shifting from GAL4-suppressive (18°C) to permissive (29°C) temperature (t_ON_). BrZ3 expression was evident 2 to 3 h following t_ON _shift (Figure 6A). We scored the resultant adult arbors for severity of ectopic arbor formed after each temperature step (Figure 6C, squares). Animal ages at t_ON _were converted into the equivalent hours after puparium formation at 25°C (hours APF^25°C^) to facilitate comparison of BrZ3 onset and offset.

Delay of BrZ3 expression until head eversion (12 h APF^25°C^) still resulted in full ectopic arbor growth, but delay of BrZ3 expression until 24 h APF^25°C ^reduced total ectopic arbor by more than 50%. Phenotypes included the typical 'comb' (16%; Figure 6C, black) and simpler arbors on the epidermis (Figure 6C, grey) such as a comb 'backbone' with no visible 'teeth' (4%), and a long microtubule-containing branch extending straight off the muscle (3%). Delay of t_ON _until 30 h APF^25°C ^produced similar low percentages of comb (26%) and intermediate (5%) phenotypes. The mildest errors (Figure 6C, white) were dendrites that extend out of the muscle groove along the posterior muscle edge or sometimes with a cluster of short fibers extending slightly past the end of the muscle groove (seen in 15 to 20% of t_ON _24 to 30 h APF^25°C ^and in 3% of control dbd arbors). No ectopic arbor formed with delay of BrZ3 expression beyond 36 h APF^25°C^.

We also used GAL80^ts ^and temperature step-down to switch off BrZ3 expression at times throughout dbd development (t_OFF_; Figure 6B) and scored the resultant adult arbors (Figure 6C, circles). Disappearance of BrZ3 following GAL80^ts ^activation was complete by 12 h at 18°C (6h^25°C^; Figure 6B). The percent of neurons with ectopic arborizations rose steeply with duration of BrZ3 exposure in early metamorphosis, reaching half maximum at 36 h APF^25°C^, but the percent with a full 'comb' rose more slowly, reaching half maximum at 43 h APF^25°C^. Most ectopic arbors were the simpler, intermediate phenotypes up to t_OFF _at 41 h APF^25°C^. More complex arbors were formed when we maintained BrZ3 for longer periods.

GAL80^ts^, C161-GAL4 > UAS-BrZ3 animals raised at 29°C until later t_OFF _times generated lower than expected percentages of large comb arbors. To see if this resulted from arbor pruning after suppression of BrZ3 expression, we took live image series of dbds from the time of t_OFF _shift through the end of arbor development. Just after t_OFF _at 43 to 44 h APF^25°C^, 62.5% of neurons had initiated a 'T' branch; most had the 'T' apex filopodial zone but with few secondary branches (n = 9 animals). Nine of ten dbds increased ectopic arbor and three new dbds showed ectopic turning onto the muscle edge at the first time point after BrZ3 removal. Although higher-order branches were difficult to see due to bleaching and dimming of GFP over time at 18°C, at later time points more neurons showed stable small ectopic arbors (n = 7 neurons, 60 to 71 h APF^25°C^), while only a few showed a reduction of the 'T' branch from either pruning or dimming (n = 3 neurons). At t_OFF _55 h APF^25°C^, all animals had one or more dbd(s) with a 'T' and secondary branches (n = 18 neurons in 7 animals), which were stable or slightly expanded at later time points (n = 9 of 14 neurons, 69 h APF^25°C^; n = 8 of 8 neurons, 75 h APF^25°C^). Overall, removal of BrZ3 during elaboration reduced subsequent ectopic arbor growth but did not lead to pruning of an existing ectopic arbor.

## Discussion

### Metamorphic remodeling of the dbd stretch receptor

**Figure 7:**
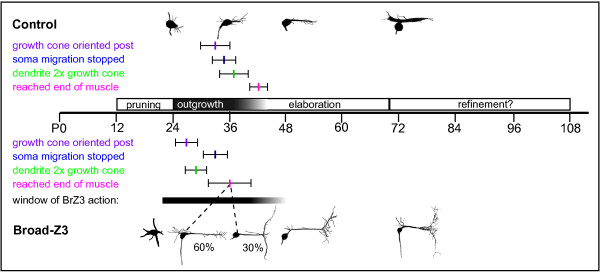
**Summary of BrZ3 outgrowth timing phenotype**. Timeline of premature outgrowth in dbd^[+BrZ3] ^in comparison to controls, in hours APF. Cartoons from representative *in vivo *z-projections. Colored bars show event times from time-lapse movies (mean ± standard deviation). Control dbd growth cones oriented to the posterior at 33 ± 3.2 h APF (n = 7), soma migration stopped at 36 ± 2 h APF (n = 6), primary dendrite elongated to twice the length of the initial growth cone by 37 ± 4 h APF (n = 4), and the posterior 'pull' indicated dendrite reached the muscle edge at 42 ± 2 h APF (n = 4). The dbd^[+BrZ3] ^growth cone oriented posteriorly by 27 ± 3 h APF (n = 7), migration stopped at 32 ± 3 h APF (n = 11), dendrite reached twice the growth cone length at 29 ± 3 h APF (n = 9), and bent at the muscle edge at 36 ± 5 h APF (n = 8). The black bar denotes the window of BrZ3 action from temperature shift experiments, adjusted for time required to express and remove BrZ3 (Figure 6). The bar extends from the 50% point along the curve for percentage of ectopic arbors after delayed BrZ3 onset, to the 50% point along the curve for percentage of ectopic arbors in BrZ3 offset experiments, with grey shading extending to the 50% point on the curve for full ectopic comb arbor.

Like a subset of its neighboring md neurons, dbd remodels during pupal development, first pruning the larval bipolar dendrite, then migrating along a persistent peripheral nerve to a new adult position, and finally building the more elaborate adult dendritic arbor [3,8,9,29] (Figure 2). Remodeling of md dendrites occurs in conjunction with the histolysis and proliferation of new epidermal cell layers, and dbd also grows onto a new adult-specific muscle substrate, the dorsal body wall muscles (Figures 1C and 2E). The dynamics of dbd dendritogenesis that we observed should be considered within the context of the muscle substrate development, as muscle and dendrite extension are likely to be closely coordinated. Both founder myoblasts and the soma of dbd migrate dorsally along the same nerve starting at 26 to 30 h APF [26,30] (Figures 2F and 3A). The founder myoblasts arrive at their final positions in the dorsal body wall by 33 h APF [30], presenting a stable cellular target for the outgrowth of dbd's dendrite (36 h APF; Figure 2E). The time period of myoblast aggregation and fusion (28 to 50 h APF) [26,30] encompasses the time window of dbd dendrite bundle elongation (Figures 2 and 7). It is possible the dbd pattern of first posterior and then anterior extension is related to the fusion pattern of the underlying myoblasts. The posterior 'pull' on each dbd neuron between 37 and 50 h APF may be correlated with myoblast attachment to the posterior segment border.

Throughout metamorphosis, all dbd dendritic growth was strictly limited to the narrow groove between adjacent dorsal abdominal muscles. When the primary dendrite reached the posterior muscle boundary, the growth cone reversed and turned back into the muscle groove (Figure 2G). Later branches throughout the arbor were also contained within the muscle groove and fasciculated rapidly (Figure 2H, I), with rapid retraction of any branches outside of the muscle groove (Figure 2J, arrowheads). These results suggest that dbd encounters a consistent signaling environment limiting growth to the muscle throughout all phases of remodeling.

While the larval form of dbd is an evolutionarily conserved, dorsal longitudinal stretch receptor [10] noted for its role in sensory feedback in crawling [31,32], the function gained by remodeling dbd into its densely fasciculated and embedded adult form is unknown. It is possible that the fasciculated shape of dbd improves the sensitivity of sensory feedback to coordinate fine motor control of adult abdominal bending movements.

### Comb-like ectopic arbor shape

The dendritic arbor of dbd is generally resistant to manipulations of dendritic shape that affect neighboring da neurons [3,33,34]. Other aberrant phenotypes for dbd are rare and mild [35]. However, ectopic expression of BrZ3 generated a dramatic transformation of the dendrite of dbd from a compact, self-fasciculated structure along muscle to a large, tree-like arbor on the epidermis. Unlike manipulations of Dscam isoforms that change self-avoidance throughout a neuron's dendritic arbor [36], BrZ3 expression does not appear to switch dbd from self-attraction to self-repulsion, because similar cellular behaviors can be seen in both experimentals and controls. Instead we think it likely that the dramatic change in morphology is due to differences in the local environment of the dendrite and how the growing dendrites interpret these new cues. In other words, the detailed comparison of control dbd and dbd^[+BrZ3] ^suggests that, in its ectopic location, the growing dendrite displays a similar program of branch formation but without fasciculation.

The stereotypical pattern of the dbd^[+BrZ3] ^ectopic arbor was reminiscent of the larval branching of class I da neurons. We think that the normal adult form of dbd is likely quite similar to a class I da neuron but that it is compacted into the space between the two muscles. The exogenous BrZ3 expression then serves to 'unpack' the adult dbd arbor onto the epidermis. The initial 'T' branch could have arisen from a path-finding error at the muscle's posterior boundary where the growth cone turned 90° instead of 180°. Branches continued to form from filopodia at the posterior boundary in both dbd^[+BrZ3] ^and controls, with dbd^[+BrZ3] ^branches completing the 'T' branch in addition to adhering back onto the preceding neurite or muscle. The interstitial filopodia in the control dbd arbor first extended perpendicular to the main arbor trunk before fasciculating, not unlike the perpendicular filopodia that form the comb 'tines' of the dbd^[+BrZ3] ^arbor. The reason that the ectopic arbor did not fasciculate may be that the epidermis was not permissive for this behavior (Figure 5I). Altogether, the branching pattern that built the fasciculated adult dbd arbor is reminiscent of the da-like branching seen in the dbd^[+BrZ3] ^ectopic arbor. This suggests that during metamorphosis, dbd follows a growth and elaboration program for its dendritic arbor that is similar to that of neighboring da neurons.

### Early phenocritical period for BrZ3 action during initial outgrowth

How does the ectopic arbor arise? To better understand the answer, we looked at the early stages of dendrite elaboration. The primary dendrite of dbd^[+BrZ3] ^extended 7 h earlier than in controls (Figure 7, green). With muscle staining, we saw that dbd^[+BrZ3] ^primary dendrites reached the posterior muscle boundary by 32 h APF, 4 h before controls began dendritogenesis (n = 6 of 8 animals; Figure 5F). Time-lapse videos also showed dbd^[+BrZ3] ^'T' branches started by 36 h APF, 6 h before control dbd posterior dendrites stabilized in length and showed the 'pull' thought to indicate attachment to muscle (Figure 7). Hence, dbd^[+BrZ3] ^dendrites reached the posterior boundary of the segment 6 to 10 h earlier than controls.

When we delayed BrZ3 expression using temperature shifts, we found that the ectopic arbor depended on BrZ3 being present during this window of premature dendritogenesis (Figure 6A, C). In normal cells outgrowth begins between 32 and 40 h APF. When BrZ3 expression was delayed until 38 h APF^25°C ^(t_ON _36 h APF^25°C ^plus 2 h for BrZ3 onset) only 9% of all neurons subsequently formed ectopic dendrites, with less than 1% forming a comb. Therefore, the delay of BrZ3 expression until the start of primary dendrite extension was not sufficient to support the later formation of an ectopic arbor, even though arbor elaboration continued for another approximately 20 h in the presence of BrZ3. In fact BrZ3 expression during the entire period of premature dendritogenesis was required to generate an ectopic arbor in the majority of dbd neurons; BrZ3 expression starting at 26 or 32 h APF^25°C ^failed to induce an ectopic arbor in 70 to 75% of dbd neurons (t_ON _24 or 30 h APF^25°C ^plus 2 h for BrZ3 onset). Thus, early BrZ3 expression coincident with the premature outgrowth seen in dbd^[+BrZ3] ^was necessary for the later ectopic branching (Figure 7).

Experiments stopping ectopic BrZ3 expression before the comb had completed development tested whether phasic expression of BrZ3 was sufficient to generate ectopic arbors (Figure 6B, C). When BrZ3 was suppressed at approximately 40 h APF^25°C^, when dbd^[+BrZ3] ^neurons first bent around the posterior muscle boundary, only 30% developed any level of ectopic arbor (t_OFF _34.5 h APF^25°C ^plus approximately 6 h for BrZ3 offset). This may reflect the limited number of dbds that had stabilized a long 'T' branch by this time. When we delayed the suppression of BrZ3 expression until after all neurons had started their 'T' branch, at approximately 47 h APF^25°C^, most dbds produced ectopic arbors (t_OFF _41 h APF^25°C ^plus approximately 6 h for BrZ3 offset). This sharp increase suggested that after the first ectopic branch was built, its subsequent stability was independent of BrZ3. Our image series following BrZ3 removal confirmed the stability of 'T' branches after disappearance of BrZ3. However, the longer BrZ3 exposure times needed to produce high percentages of highly branched ectopic combs suggested that BrZ3 may play some role in the stabilization of the higher-order ectopic arbor branches. Image series showed that pruning of secondary branches did occur after BrZ3 removal (n = 8 neurons), but pruning was similar in extent to that observed in dbd^[+BrZ3] ^with continuous expression of BrZ3 (n = 7). Instead, most mild phenotypes in the image series developed from smaller 'T' scaffolds with fewer filopodia (n = 14 neurons). Late-developing ectopic arbors in particular were often too small to extend past the muscle substrate, in conditions of either phasic or continuous BrZ3. This could reflect stronger inhibition for growth away from the muscle onto the epidermis as these tissues continue to mature.

Overall, these results reveal that BrZ3 expression during the initial phase of primary dendrite outgrowth was sufficient to generate an ectopic branch onto the epidermis and that the extent of that ectopic arbor, though, depended on continuing expression of BrZ3. It suggests that the ectopic arborization is due to a heterochrony between the neuron and its muscle target.

### Cellular interactions involved in dendritic errors

There are several possible interpretations of the relationship between the premature outgrowth and ectopic arbor components of the dbd^[+BrZ3] ^dendritic phenotype. We favor the model that exogenous BrZ3 cell-intrinsically promoted premature outgrowth, which led to the secondary effect of a misplaced arbor. BrZ3 could promote outgrowth at the growth cone by shifting actin treadmilling towards branch extension, or through promoting some aspect of microtubule-associated branch stabilization; such local effects at the growth cone could be mediated by a transcriptional target of Broad itself, heterophilic interaction with other BTB/POZ transcription factors implicated in dendritic branching [34,37,38] or with BTB/POZ actin-binding proteins [39,40]. BrZ3 could also act further upstream, on outgrowth timing. For example, exogenous BrZ3 could shift dbd to a growth phase usually induced at higher levels of ecdysone. It is possible that BrZ3 expression could lead to a premature rise in the ecdysone- and Broad-responsive heterochronic microRNA let-7-C complex [41] leading to premature loss of abrupt function. This pathway has been shown to be important for metamorphic neuromuscular junction maturation [42,43], both *let-7-C *and *abrupt *are expressed in dbd, and an *abrupt *mutant caused the only other known dbd branching phenotype (data not shown and [35], but see [44]).

Early extension of dendrites in dbd^[+BrZ3] ^may have decoupled the timing of neurite extension from the development of the underlying muscle. Both control dbd and dbd^[+BrZ3] ^extended primary dendrites along immature myoblasts that had not yet completed aggregation and fusion [26,30] (Figures 2E and 5E-G). The extending dendrites of control dbd neurons reached the posterior end of the muscle after the latter had established attachments with the segment border, but dbd^[+BrZ3] ^dendrites reached the posterior muscle boundary 6 to 10 h earlier, before these attachments were made. This correlation suggests that the newly formed muscle-epidermal junction may produce a repulsive signal that causes the dendritic growth cone to fold back on itself. The prematurely extending growth cones of the dbd^[+BrZ3] ^neurons, though, reach the end of the myoblasts before the epidermal junction is established and so no repulsive signal is present and they grow off the muscle and onto the epidermis.

Alternatively, interference with neuron-muscle interaction could be the proximal cause for both the early outgrowth and the misplaced growth onto the epidermis seen with BrZ3 expression. Dbd is closely apposed with its developing myoblast substrate throughout metamorphosis. These myoblasts could provide important growth inhibition signals to limit both the onset and extent of dbd growth. BrZ3 could disrupt the ability of dbd to respond to these inhibitory signals, leading to both the outgrowth during migration and past the muscle.

### Insight into endogenous BrZ3 function

While global CNS phenotypes for null Broad alleles have been long established [19], no clear cell-autonomous function for BrZ3 has been identified in neurons. A weak BrZ3 mutant allele, 2Bc^2^, showed deficient dendritic arborization in flight motor neurons that was rescued by ubiquitous BrZ3 expression, but this was shown to be a non-cell-autonomous effect [45]. Other than the present study, the only selective expression of BrZ3 to date used a pdf-GAL4 driver and found expansion of the dorsal projections in small LNv neurons toward the midline [22]. However, endogenous BrZ3 is not found in either the flight motor neurons or the remodeling LNv neurons. Similarly, we did not detect endogenous Broad in dbd with a Broad-Core antibody (14 to 40 h APF, n ≥ 5 for each time point every 2 h), and Broad-Core RNA interference (RNAi) did not qualitatively change the dbd arbor (n = 11 neurons in 6 animals).

Overall, the evidence points to lack of Broad during the process of remodeling larval neurons to generate the adult arbors. In contrast, BrZ3 is expressed in all newly developing interneurons, whether their initial outgrowth and branching progress during embryonic or metamorphic development [24]. This finding raises the intriguing question, in remodeling cells, how does the post-pruning regrowth differ from the process of initial outgrowth? Continued comparison of larval and pupal development of the persistent md neurons could offer key insights into the differences between *de novo *and regenerative neuronal growth.

Close examination of BrZ3 action in dbd suggests a possible specific role in promoting or coordinating the timing of outgrowth that could point to the role of BrZ3 in CNS interneurons. That the dbd phenotype was specific to the BrZ3 isoform is consistent with the unique developmental profile of BrZ3 in the CNS; all adult-specific interneurons showed robust BrZ3 expression during metamorphic development, while expression of BrZ1 and BrZ4 were much more restricted [22]. The effects of BrZ3 in dbd were limited to a narrow temporal window early in metamorphosis, as the primary dendrite extended and interacted with its target. BrZ3 expression in CNS interneurons is limited to a similar developmental window, during sprouting and initial target selection, and is removed before arbor elaboration ([22] and Scott and Truman, in preparation). This temporal correlation suggests that endogenous BrZ3 may play a role in early neurite outgrowth and interaction with initial targets, similar to its action in dbd.

## Conclusions

Unlike the simple arbor of the larval stage, the adult dendritic arbor of dbd is a highly branched structure whose branches self-fasciculate to form a compact dendritic bundle. The ectopic expression of BrZ3 in this cell causes a premature extension of its growth cone, resulting in dendrites that extend beyond their normal muscle substrate and onto the epidermis where they form a comb-shaped, ectopic arbor. The experiments involving the temporal control over BrZ3 expression show that the most important time for BrZ3's effects is early in growth cone extension and not when the neuron is actually growing over the epidermis. The BrZ3 expression appears to cause a heterochronic advance in the outgrowth of the cell, bringing it in contact with the epidermis prior to the arrival of its normal muscle substrate. Our quantitative data suggest that the new ectopic arbor is due primarily to the 'unpacking' of the normally fasciculated arbor onto the epidermis. These data suggest that the nature of their local environment (muscle versus epidermis) can change dendrite behavior from self-adhesion to self-avoidance, and resulting in a dramatically different dendritic form.

## Materials and methods

### Fly stocks and staging

The GAL4 driver *C161-GAL4 *expresses in a subset of dorsal md sensory neurons, including dbd [3,27]. Control neurons were imaged in animals having the genotype *UAS-mCD8::GFP*; *C161-GAL4/TM3Sb *or *UAS-mCD8::GFP*; *C161-GAL4*, *UAS-mCD8::GFP/TM6b *[27]. The morphology of dbd and the developmental timing of arbor growth were identical in the two genotypes (data not shown). To express Broad in dbd, *UAS-mCD8::GFP*; *C161-GAL4*, *UAS-mCD8::GFP/TM6b *flies were crossed with *UAS-BrZ3*, *UAS-BrZ1 *and *UAS-BrZ4 *separately [46]. To remove Broad, we used a UAS-Broad-Core RNAi (a generous gift from Xiaofeng Zhou and Lynn Riddiford). Animals were collected at white puparium formation and maintained in a Petri dish with moist filter paper at 25°C until use, with the exceptions of RNAi animals, which were raised at 29°C. All staging is denoted as hours APF.

### Phenocritical period experiments

To establish the phenocritical period(s) of BrZ3 action, we used a temperature-sensitive GAL80 (GAL80^ts^), a suppressor of GAL4, to reversibly inhibit GAL4 > UAS-BrZ3 expression [47] in flies of the genotype *GAL80^ts^/+; UAS-mCD8::GFP/+; GAL4^C161^/UAS-BrZ3*. These flies were placed at initial temperature at L3 then staged at P0. For comparison, ages at the time of temperature step-ups and step-downs were converted to units of the equivalent hours APF at 25°C (h APF^25°C^). Relative to 25°C, pupal development is half as fast at 18°C, and accelerated approximately 1.15-fold at 29°C (data not shown and [48]).

### Live imaging

For single time-point images, pupae were dissected out of their puparial case and mounted under a coverslip in a small drop of halocarbon oil in an imaging chamber. Z-stacks of segments A2 to A5 were acquired using a MRC-600 or Radiance 2000 confocal microscope, 40× and 60× objectives, and LaserSharp acquisition software (Bio-Rad, Hercules, CA, USA). For time-lapse series, each animal was dissected out of its puparial case and mounted in a humidified imaging chamber between an oxygen-permeable membrane and a coverslip [3,49]. Time-lapse z-stacks of segments A2 to A4 were acquired every 30 minutes with the Bio-Rad Radiance 2000 System, using a Mai Tai laser (Spectra-Physics, Fremont, CA, USA) set at 905 nm. For temperature shift image series, specimens were stored in humidified, temperature-controlled incubators between time points. Temperature during imaging ranged between 21 and 26°C. All measurements were made from live images.

### Immunohistochemistry

The abdominal body wall was dissected, fixed in 4% formaldehyde for 30 minutes, and blocked with 1% normal donkey serum (Sigma, St Louis, MO, USA) at room temperature before overnight incubations at 4°C in primary, then secondary antibodies in phosphate buffered saline with 0.3% Triton-X100 (PBS-TX; Sigma). All washes between steps used PBS-TX. Tissues were dehydrated in an ascending EtOH series, cleared in xylene and mounted in DPX (Sigma). Primary antibodies were: rat anti-CD8 (1:1,000; Caltag Laboratories, Burlingame, CA, USA), mouse 22C10 anti-Futsch (1:200 to 1:500; Developmental Studies Hybridoma Bank), mouse Broad-Core (1:500) [20] and rabbit BrZ3 (1:3,000; a gift from J-A Lepesant and C Antoniewski) [16]. Secondary antibodies included: AlexaFluor 488 goat anti-rat (1:500; Molecular Probes, Eugene, OR, USA), and Texas Red donkey anti-mouse or anti-rabbit (1:500; Jackson ImmunoResearch, West Grove, PA, USA), or Texas Red-X phalloidin F-actin stain (1:200; Molecular Probes). All Gal80^ts ^temperature shift preparations were incubated in primary antibody for 3 days and in secondary for 2 days. Fixed tissue preparations were imaged using either the confocal microscopes described above or a Zeiss LSM 510 (Jena, Germany), with 40× and 63× objectives.

### Image analysis

Image z-stacks and time series were assembled in NIH ImageJ [50] and adjusted for brightness and contrast. Arbor measurements are averages of at least three measurements per neuron. Scoring of aberrant arbor characteristics for temperature shift experiments was repeated twice by a single, blinded observer. No individual temperature category had a disproportionate number of preparations excluded because of disruptions (6 to 15%). All statistical analysis was performed using SPSS software (Chicago, IL, USA) with curve fitting in DeltaGraph (Red Rock Software, Salt Lake City, UT, USA) and curve analysis in Prism (GraphPad, La Jolla, CA, USA).

## Abbreviations

APF: after puparium formation; BrZ3: Broad isoform Z3; CNS: central nervous system; dbd: dorsal bipolar dendrite neuron; GAL80^ts^: temperature-sensitive GAL80; GFP: green fluorescent protein; md: multidendritic sensory neuron; RNAi: RNA interference; UAS: upstream activation sequence.

## Competing interests

The authors declare that they have no competing interests.

## Authors' contributions

DWW helped conceive of the project, gave advice during its execution, and contributed to the writing of the manuscript. JAS participated in the design of the study, carried out the experiments, performed the statistical analysis and drafted the manuscript. JWT participated in the design of the study and helped to draft the manuscript. All authors read and approved the final manuscript.

## Supplementary Material

Additional file 1 **Supplemental video 1 - migration and early outgrowth of dbd and ddaE**. Z-projections of time-lapse two-photon series of control dbd from 28 to 45 h APF from Figure 4A, showing migration of dbd and ddaE in opposite directions along the nerve, growth cone turning and initial outgrowth. Z-stacks were taken every 30 minutes, and the animal was re-centered under the microscope at 43 h APF. Click here for file

Additional file 2 **Supplemental video 2 - metamorphic elaboration of the dbd arbor**. Time-lapse two-photon image series of dendritogenesis in two control dbd neurons from 33.5 to 66 h APF, segments A2 and A3. The right neuron has an early anterior primary dendrite that is removed. The left neuron shows posterior 'pull' more clearly, between 37 and 38 h APF. Most filopodial activity ended by 51 h APF. Z-stacks were taken every 30 minutes, and the animal was re-centered under the microscope at 46 h APF. Click here for file

Additional file 3 **Supplemental video 3 - outgrowth phenotypes with ectopic BrZ3 expression**. Time-lapse two-photon series of dbd^[+BrZ3] ^from 25 to 40 h APF showing premature outgrowth of the primary dendrite and initial ectopic branch formation. Z-stacks were taken every 30 minutes. Click here for file

Additional file 4 **Supplemental video 4 - branches folding back into fasciculation**. Time-lapse two-photon image series of dbd^[+BrZ3] ^from 33 to 50 h APF. Flexing in the preparation caused the dbd neuron to fall out of the recorded stack, which gave a clear view of foldback branches. Z-stack projections were taken every 30 minutes, and the animal was re-centered under the microscope at 46 h APF. Click here for file

Additional file 5 **Supplemental video 5 - **h**igher-order branching and pruning on the ectopic arbor**. Complete time-lapse two-photon image series from 50 to 64 h APF of dbd^[+BrZ3] ^from Figure 5I showing higher-order branching and removal of branches that attempted to fasciculate along the ectopic arbor and to grow along the top edge of the muscle. Z-stack projections were taken every 30 minutes. Click here for file
